# Influence of Additive Manufactured Scaffold Architecture on the Distribution of Surface Strains and Fluid Flow Shear Stresses and Expected Osteochondral Cell Differentiation

**DOI:** 10.3389/fbioe.2017.00006

**Published:** 2017-02-10

**Authors:** Wim J. Hendrikson, Anthony J. Deegan, Ying Yang, Clemens A. van Blitterswijk, Nico Verdonschot, Lorenzo Moroni, Jeroen Rouwkema

**Affiliations:** ^1^Department of Tissue Regeneration, MIRA Institute for Biomedical Technology and Technical Medicine, University of Twente, Enschede, Netherlands; ^2^Institute for Science and Technology in Medicine, School of Medicine, Keele University, Stoke on Trent, UK; ^3^Complex Tissue Regeneration Department, MERLN Institute for Technology Inspired Regenerative Medicine, University of Maastricht, Maastricht, Netherlands; ^4^Department of Biomechanical Engineering, MIRA Institute for Biomedical Technology and Technical Medicine, University of Twente, Enschede, Netherlands; ^5^Orthopaedic Research Laboratory, Radboud Nijmegen Medical Centre, Nijmegen, Netherlands

**Keywords:** FEA, CFD, additive manufacturing, scaffold architecture, tissue engineering, cell differentiation

## Abstract

Scaffolds for regenerative medicine applications should instruct cells with the appropriate signals, including biophysical stimuli such as stress and strain, to form the desired tissue. Apart from that, scaffolds, especially for load-bearing applications, should be capable of providing mechanical stability. Since both scaffold strength and stress–strain distributions throughout the scaffold depend on the scaffold’s internal architecture, it is important to understand how changes in architecture influence these parameters. In this study, four scaffold designs with different architectures were produced using additive manufacturing. The designs varied in fiber orientation, while fiber diameter, spacing, and layer height remained constant. Based on micro-CT (μCT) scans, finite element models (FEMs) were derived for finite element analysis (FEA) and computational fluid dynamics (CFD). FEA of scaffold compression was validated using μCT scan data of compressed scaffolds. Results of the FEA and CFD showed a significant impact of scaffold architecture on fluid shear stress and mechanical strain distribution. The average fluid shear stress ranged from 3.6 mPa for a 0/90 architecture to 6.8 mPa for a 0/90 offset architecture, and the surface shear strain from 0.0096 for a 0/90 offset architecture to 0.0214 for a 0/90 architecture. This subsequently resulted in variations of the predicted cell differentiation stimulus values on the scaffold surface. Fluid shear stress was mainly influenced by pore shape and size, while mechanical strain distribution depended mainly on the presence or absence of supportive columns in the scaffold architecture. Together, these results corroborate that scaffold architecture can be exploited to design scaffolds with regions that guide specific tissue development under compression and perfusion. In conjunction with optimization of stimulation regimes during bioreactor cultures, scaffold architecture optimization can be used to improve scaffold design for tissue engineering purposes.

## Introduction

In the field of tissue engineering, a scaffold can be used to provide a temporary or permanent support structure for cells to form a tissue. In the specific case of musculoskeletal regeneration, a general tenet is the need for a scaffold design capable of withstanding load-bearing forces. From literature, it is known that scaffold architecture influences the apparent stiffness of the scaffold (Moroni et al., 2006a,b). The scaffold design can, for instance, be tailored to match the mechanical properties of native tissues (Moroni et al., 2006a). However, since changing scaffold architecture will change stress and strain distributions within the scaffolds during bioreactor cultures under compression and perfusion conditions, it is important to understand the relationship between scaffold design and biophysical signals (Guilak et al., 2014).

It is becoming clear that cells are sensitive to deformations within their environment and adjust their behavior accordingly (Brown and Discher, 2009; Wang et al., 2009; Buxboim et al., 2010). A number of mechano-regulation theories exist that relate the biophysical stimuli to specific tissue formation (Pauwels, 1960; Carter et al., 1988; Prendergast et al., 1997; Claes and Heigele, 1999). By predicting the stress and strain distribution in a scaffold using finite element analysis (FEA), and coupling this with cell differentiation and tissue formation (Byrne et al., 2007; Checa and Prendergast, 2010; Milan et al., 2010; Sandino and Lacroix, 2011), these theories can be used to optimize scaffold design parameters, such as the type of biomaterial, porosity, and architecture. Most mechano-regulation theories reported in literature are based on a model proposed by Prendergast (Lacroix and Prendergast, 2002). With the aid of FEA, this mechano-regulation theory has been used extensively to predict bone healing in multiple settings (Isaksson et al., 2006; Checa et al., 2011) and also to investigate the effect of scaffold properties on tissue formation in hypothetical (Byrne et al., 2007) and computer aided design (CAD)-based models of additive manufactured scaffolds (Olivares et al., 2009) in a tissue engineering setting.

Byrne et al. (2007) used the mechano-regulation theory of Prendergast to investigate the effect of scaffold design on tissue formation. They showed that the Young’s modulus, porosity, and dissolution rate of the scaffold were influential on cell differentiation and tissue formation in the scaffold. Olivares et al. (2009) investigated the effects of porosity, and pore shape and size on cell differentiation stimulus values using two variations of regular scaffold architectures. This study also showed a strong relationship between pore size and porosity, and cell differentiation stimulus, but acknowledged the pore shape to be as important.

Even though it has been shown that pore shape within a scaffold has a large effect on stress and strain distribution, a CAD-based finite element model (FEM) is generally not able to capture the stress and strain distribution in actual scaffolds due to variations in scaffold architecture inherent to most fabrication processes (Hendrikson et al., 2014). This means that FEMs should be based on micro-CT (μCT) scans of actual scaffolds to obtain accurate representations of the pore shapes to investigate the effect of scaffold architecture on cell differentiation stimulus.

Previous studies on the prediction of tissue formation based on μCT scans have been performed for salt-leached scaffolds (Checa and Prendergast, 2010; Milan et al., 2010; Sandino and Lacroix, 2011). It proved to be valuable in showing the influence of pore shape on stress and strain distributions. Yet, the salt leaching fabrication process makes it difficult to control pore size, shape, and porosity and obtain reproducible scaffolds, thereby rendering it a suboptimal method to investigate the influence of scaffold architecture on cell behavior.

Additionally, mechano-regulation models reported in literature are based on volumetric strains. However, at the initial stage of cell differentiation, the cell is attached to the scaffold surface. Since cells can only sense deformations up to a small distance (Sen et al., 2009; Buxboim et al., 2010), a model using surface strains instead of volumetric strains will provide a better representation of the initial signals that cells seeded on a scaffold will experience. We have shown previously for a single scaffold architecture that the predicted strain for the scaffold material volume is considerably higher than the predicted strain for the scaffold surface on which cells are attached (Hendrikson et al., 2014), indicating that most current models overestimate the mechanobiological signals that cells experience.

In this study, additive manufacturing (AM) was used to produce scaffolds with four different scaffold designs. AM ensures a high controllability and reproducibility of the scaffold architecture, so that fiber diameter, fiber spacing, and layer thickness were constant across the different designs. By changing the angle of layer deposition and shifting layers laterally, different architectures were obtained. Most notably, designs where the layers were not shifted laterally contained crossing fibers at the same position in each layer. This created vertical supporting columns for the load. In designs where layers were shifted laterally, the locations of crossing fibers in successive layers varied, resulting in the absence of supporting columns. In order to investigate the effect of scaffold architecture on stress and strain distribution and subsequently cell differentiation stimulus prediction, μCT-based models of the scaffolds were prepared, and stress and strain distributions within the scaffolds were predicted using computational fluid dynamics (CFD) and FEA.

The goal of this study was to investigate the influence of additive manufactured scaffold architecture on the distribution of surface strains and fluid flow shear stresses within the scaffold, and subsequently the expected cell differentiation. The results show a distinct effect of the scaffold architecture on surface strains and fluid flow shear stresses under mechanical compression and imposed fluid flow. As reported before, the octahedral shear strain magnitudes exceed the surface shear strain magnitudes, which are reflected in the cell differentiation stimulus values on the scaffold surface. The results of the study show that regions of the scaffold could be designed favoring specific cell differentiation stimuli. Coupling with biophysical loading regimes *a priori in silico* could accelerate the design of scaffolds and optimize the loading regimes for tissue engineering purposes.

## Materials and Methods

### Scaffold Fabrication

Scaffolds were fabricated by a bioscaffolder (SYSeng, Germany), as previously described (Hendrikson et al., 2014). Briefly, granules of the block copolymer 300PEOT55PBT45 (PolyVation B.V.) were heated to 190–200°C. An applied nitrogen pressure of 5 bar and an auger screw rotating at 200 RPM extruded the molten polymer through a 250 µm inner diameter needle (DL technology) on a stationary platform. Scaffolds were fabricated through layer-by-layer deposition, in which the angle between layers, fiber spacing, and layer thickness can be set. A scaffold block of 30 mm × 30 mm × 2.1 mm was created for four different architectures (Figure S1 in Supplementary Material). All architectures had a fiber spacing of 1,000 µm and a layer thickness of 150 µm. The 0/90 architecture had a 90° angle between successive layers, while the 0/45 had a 45° angle between layers. The 0/90 offset and 0/45 offset had the same parameters as the 0/90 and 0/45 architectures, respectively, but the layers with the same fiber direction were shifted 500 µm in the lateral direction of subsequent layers creating a staggered architecture. Cylindrical scaffolds of 8 mm in diameter were obtained by using a biopsy puncher (Miltex). The actual fiber diameter, spacing, and layer height were measured from μCT images in the freeware program MicroView 2.1.2. Five measurements were taken at different random locations for each scaffold, and the average and SD were calculated. The porosity was determined for the whole scaffold and the selection of the scaffold used to make the model. The porosity was calculated as the ratio of the material volume to the total volume, as determined by MicroView.

### μCT Scanning

Scaffolds were scanned with a Scanco μCT 40 (Scanco Medical, Sweden) in a custom designed holder with which a fixed compression can be applied, as previously described (Baas and Kuiper, 2008). Briefly, a scaffold was placed in a cylinder and compressed by a piston that was fixated in position by two screws. The compression was displacement controlled by using a Bose Electroforce 3200. Scaffolds were scanned with or without a 10% compression applied. The energy used was 55 kV, with an intensity of 145 µA, an integration time of 1.5 s, and 1,000 projections per 180°. Voxel sizes were 10 µm × 10 µm × 10 µm for all scans performed. Scans were exported as DICOM for further processing to FEA and CFD meshes.

### Meshes for FEA and CFD

In order to limit the number of finite elements and therefore to be able to compare the simulated deformation with the compression-scanned deformation, all scans of the uncompressed scaffolds were downsampled to a voxel size of 20 µm × 20 µm × 10 µm. The result was segmented for the material part to obtain a surface mesh of the whole scaffold. Remeshing was applied to improve the quality of the mesh and reduce the number of finite elements in order to simulate the whole scaffold with FEA.

In order to investigate the influence of scaffold architecture on tissue stimulus predictions on the scaffold surface, a region-of-interest was selected from the center of the scaffold consisting of at least two pores in the lateral directions and nine layers in the vertical direction. Fluid flow inlets were added on top and below the scaffold by expanding the selection in the vertical direction with a 100 µm void on both sides. The results were exported as DICOM to Mimics (Materialise) and segmented with the material as scaffold and the void as fluid volume. Remeshing was applied to improve the quality of the meshes.

### FEA and CFD Setup

Linear elastic and isotropic material properties of 300PEOT55PBT45 were taken from literature, for which a bulk Young’s modulus of 88 MPa (Sakkers et al., 1998) and a Poisson’s ratio of 0.48 (Moroni et al., 2006a) were considered. An unconfined compression was simulated in Marc Mentat 2014 (MSC software). The FEM was placed between two dies from which the top die applied the 10% compression. The bottom nodes of the model were fixed in the loading direction. Additionally, two corner nodes at the bottom were fixed in either the *X*-direction or the *Y*-direction along the *Y*-axis or *X*-axis, respectively, to prevent rotation of the FEM while allowing for lateral expansion.

The boundary conditions for CFD, fluid inlet, zero pressure outlet, and no slip at the boundary wall were assigned to the fluid volume mesh in Mimics simulating a close fit of the scaffold in a bioreactor. CFD simulations were performed in Fluent 13.0 (ANSYS) with a fluid velocity of 100 µm/s. The fluid was modeled as an incompressible Newtonian fluid based on culture medium properties, with a density of 1,000 kg/m^3^ and a viscosity of 1.45 × 10^−3^ Pa s (Bacabac et al., 2005).

### Simulations

#### Validations

For the validation between the scanned and simulated compression of the scaffold, the deformed mesh of the FEM from the complete scaffold was exported. In Mimics, the meshes were superimposed for their fit and visually assessed for their similarities.

#### Differentiation Stimulus

A custom-made MATLAB script identified the shared surface faces of the CFD and FEA models. A Fortran subroutine with user code was written and executed during an FEA simulation by Marc Mentat to calculate the surface strains; the strains of the element face lying on the surface of the material FEM (Hendrikson et al., 2014). The octahedral shear strains were also calculated for the same elements. The surface and octahedral shear strain were defined as

(1) $$\gamma_{\text{surf}}=\frac{\varepsilon_{1}-\varepsilon_{2}}{2}$$

with ϵ_1_ and ϵ_2_ the principal strains of the element face on the surface (Figure S2A in Supplementary Material).

(2) $$\gamma_{\text{oct}}=\frac{2}{3}\sqrt{{\left(\varepsilon_{\text{I}}-\varepsilon_{\text{II}}\right)}^{2}+{\left(\varepsilon_{\text{II}}-\varepsilon_{\text{III}}\right)}^{2}+{\left(\varepsilon_{\text{III}}-\varepsilon_{\text{I}}\right)}^{2}}$$

with ϵ_I_, ϵ_II_, and ϵ_III_ the principal strains at the integration point of the same element (Sandino and Lacroix, 2011) (Figure S2B in Supplementary Material).

Identification of the shared element faces enabled the visualization of the CFD simulation results on the material model in Marc Mentat. CFD simulation results were used to link the biophysical stimuli with cell differentiation throughout the FEA simulation through an adapted version of the mechano-regulation theory of Prendergast (Lacroix and Prendergast, 2002).

(3) $$S=\frac{\gamma }{a}+\frac{\tau }{b}\text{.}$$

The fluid flow was substituted with the fluid wall shear stress τ (Sandino and Lacroix, 2011) as calculated by Fluent, with γ either the surface shear strain or the octahedral shear strain. Constants *a* and *b* were chosen as 0.0375 and 0.010 Pa, respectively, as reported in literature (Olivares et al., 2009; Sandino and Lacroix, 2011). The cell stimulus value (*S*) thresholds used for cell differentiation were 0 ≤ *S* < 0.001 for resorption, 0.001 ≤ *S* < 1 for bone, 1 ≤ *S* < 3 for cartilage, 3 ≤ *S* < 6 for fibrous tissue (FBT), and *S* ≥ 6 for necrosis (Olivares et al., 2009; Sandino and Lacroix, 2011).

## Results

### Scaffold Characterization

The experimentally determined fiber spacing and layer thickness was similar for scaffolds of all prepared geometries (Table 1). The measured fiber diameter was slightly smaller for the 0/45 offset geometry, which could be due to the larger unsupported gaps these fibers have to span in this conformation, leading to local variations in the fiber thickness. A slight variation in the porosity of the printed scaffolds was observed, ranging from 64% for the 0/45 offset geometry to 74% for the 0/45 geometry. The porosity of the FEMs correlated well with the printed scaffolds.

**Table 1:** **Scaffold measurements based on μCT scans**.

Architecture	Fiber diameter (μm)	Fiber spacing (μm)	Layer thickness (μm)	Porosity scaffold % (porosity model %)
0/45	194 ± 11	992 ± 21	151 ± 3	74.4 (72.6)
0/45 offset	214 ± 5	982 ± 21	151 ± 4	64.1 (63.6)
0/90	212 ± 13	990 ± 20	150 ± 13	66.3 (62.4)
0/90 offset	222 ± 13	982 ± 13	151 ± 3	68.5 (67.0)

### Model Validation

**Figure 1:**
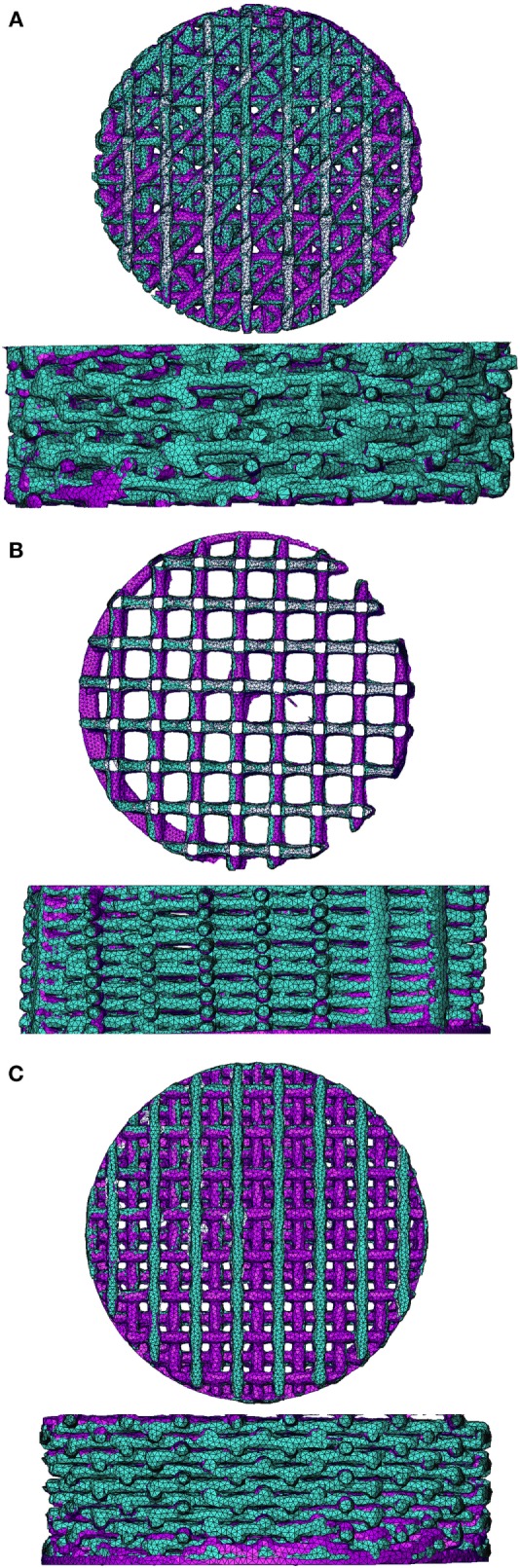
**Superimposed image of compression-scanned (pink) and compression-simulated (green) scaffolds for 0/45 offset (A), 0/90 (B), and 0/90 offset (C) architectures shows good overlap**. The 0/45 architecture is omitted due to lack of pre- and post-compression scans of the same scaffold.

To validate the FEA simulation, a FEM was made for both the compressed and non-compressed μCT-scanned scaffold using the same methodology. The compression-simulated FEM was exported and superimposed on the compression scanned FEM in Mimics. Ideally, the compression-simulated FEM would overlap perfectly with the FEM prepared based on the compressed μCT-scanned scaffold. A visual fit of the simulated and compressed model for the different architectures is shown in Figure 1 for the 0/90, 0/90 offset, and 0/45 offset architectures. There is a high degree of overlap between the simulated and compressed models for the different architectures, showing that the FEA simulation replicates the compression of the scaffolds well. Small differences were mainly seen in the compression direction.

### FEA Simulation

**Figure 2:**
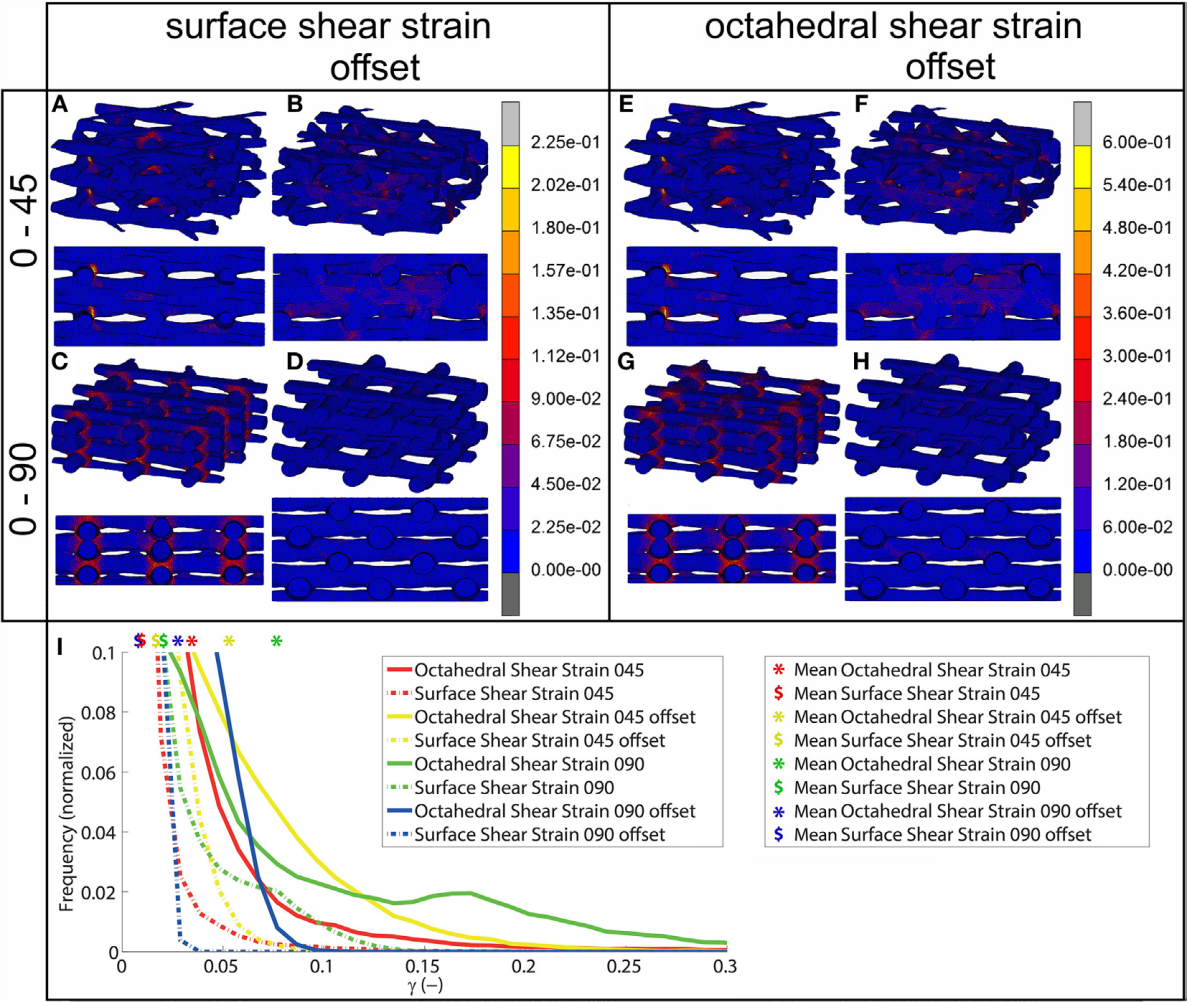
**Shear strain distribution of 0/45 (A,E), 0/45 offset (B,F), 0/90 (C,G), and 0/90 offset (D,H) architectures for surface shear strain (A–D) and octahedral shear strain (E–H) and histogram of the shear strains developed (I) after 10% compression applied**. Both trimetric and side views are shown. For a better appreciation of the strain profile in the 0/90 offset geometry, the reader is referred to Figure S3 in Supplementary Material, which shows the strain distributions using a different color scale.

Using FEA simulation, FEMs of the different architectures were subjected to a compressive strain of 10%. For the 0/90 and 0/45 scaffolds, the strain was confined to the area where the fibers cross and minimally extends along the fiber. In the 0/90 offset and 0/45 offset, the strain also developed at the crossing of the fibers but extended further (Figure 2; Figure S3 in Supplementary Material). The fraction of the surface area affected by the mechanical compression was, therefore, higher for the offset architectures. For the 0/90 architecture, the strain magnitudes were higher compared to its offset architecture counterpart while the strain magnitudes for 0/45 offset were higher than the 0/45 architecture (Table 2; Figure 2I). For all architectures, the average strain magnitudes were higher for the octahedral shear strain than for the surface strain (0.076 and 0.021, respectively, for 0/90, 0.028, and 0.0096, respectively, for 0/90 offset, 0.035 and 0.011, respectively, for 0/45, and 0.053 and 0.017, respectively, for 0/45 offset).

**Table 2:** **Average mechanical shear strains and fluid flow shear stresses**.

Architecture	Surface shear strain (–)	Octahedral shear strain (–)	Fluid wall shear stress (mPa)
0/45	0.0110	0.0350	4.4
0/45 offset	0.0166	0.0531	5.6
0/90	0.0214	0.0756	3.6
0/90 offset	0.0096	0.0276	6.8

### CFD Simulation

**Figure 3:**
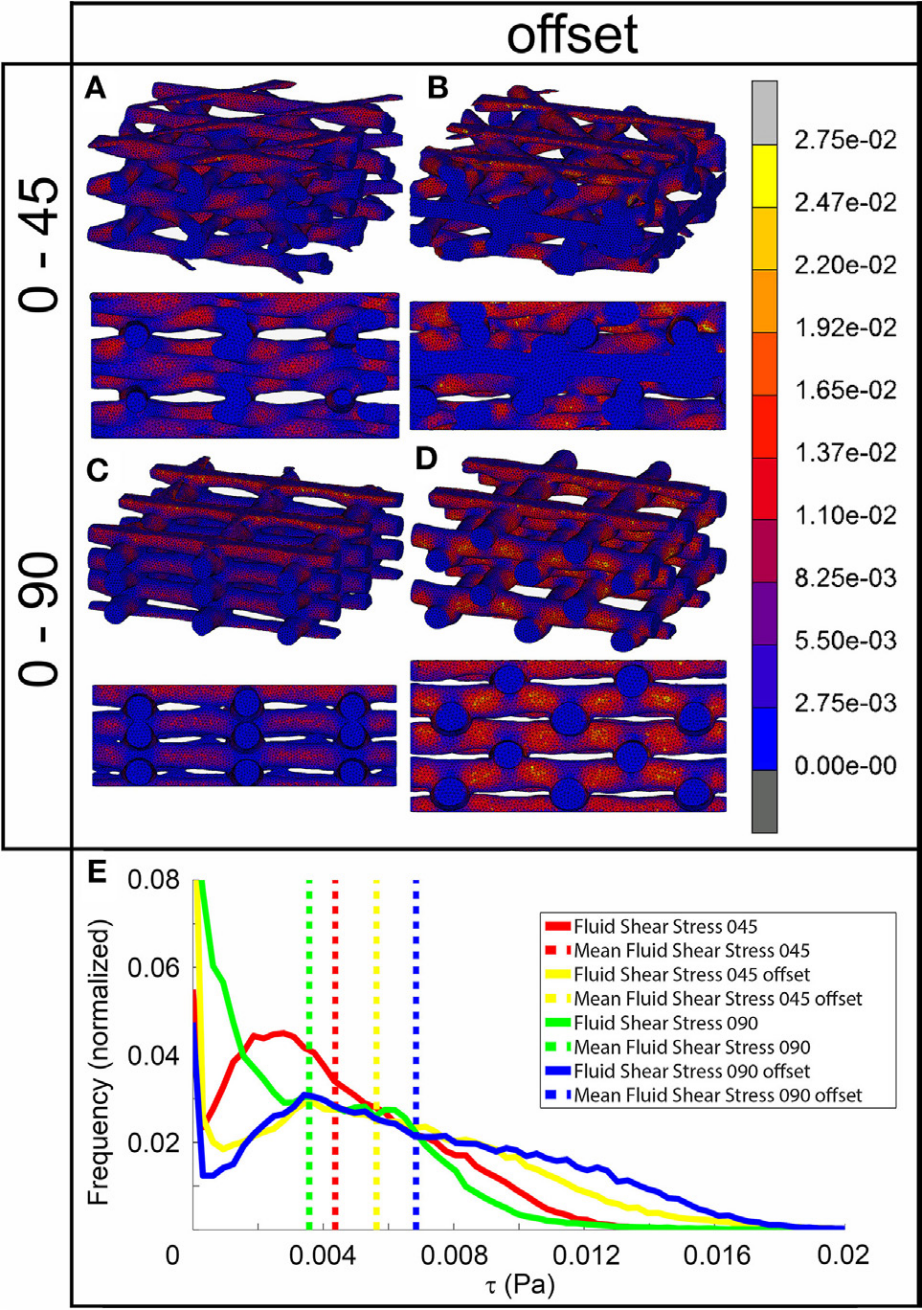
**Fluid shear stress distribution of 0/45 (A), 0/45 offset (B), 0/90 (C), and 0/90 offset (D) architectures and histogram of the fluid shear stresses developed (E) with a 100 µm/s inlet fluid velocity**. Both trimetric and side views are shown.

The fluid wall shear stress magnitude was highest on the lateral sides of the fibers in all architectures (Figure 3). The mean magnitude of shear stress was highest for the architectures with the offset (Table 2), while it was the lowest for the 0/90 scaffold. The shear stress histogram (Figure 3E) showed a higher frequency of higher shear stress magnitudes for the 0/45 offset (yellow) and 0/90 offset (blue), while it showed a higher frequency of the lower shear stress magnitudes for the 0/90 (green) and 0/45 (red) scaffold. In the 0/90 scaffold, the top fibers shielded the lower fibers from shear stress along the top side of the fiber. For the 0/90 offset scaffold, the shielding effect was reduced. The shielding could be seen to a lesser extent in the 0/45 and 0/45 offset scaffolds. Additional models using different fluid flow velocities showed that the magnitudes of the shear stress depended on fluid flow velocity, but that the shear stress distributions were independent on inlet fluid velocities (data not shown).

### Cell Differentiation Stimulus Prediction on the Surface

**Figure 4:**
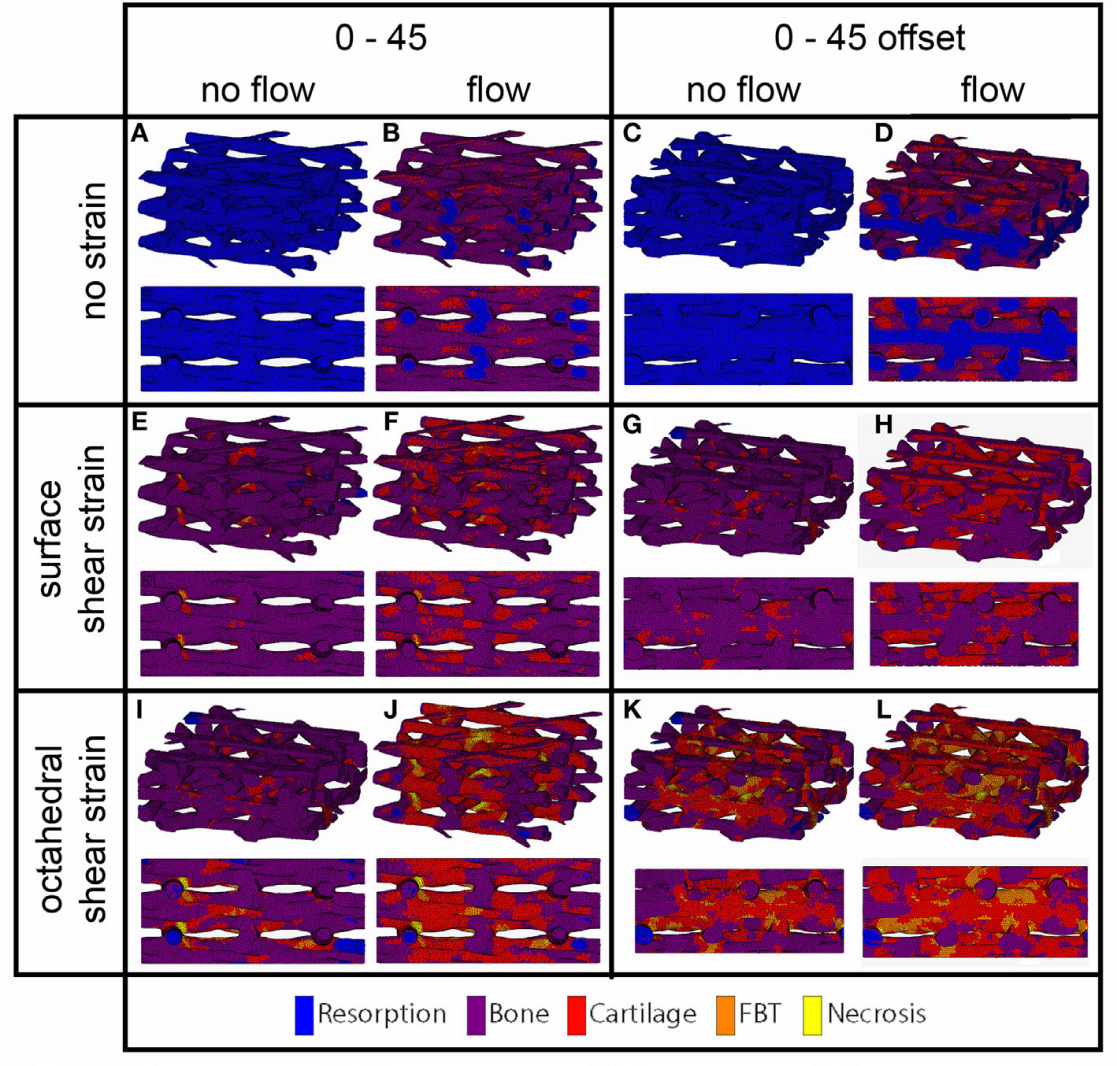
**Cell differentiation stimulus distribution for 0/45 (A,B,E,F,I,J) and 0/45 offset (C,D,G,H,K,L) architectures based on fluid flow alone (B,D), surface shear strain alone (E,G), octahedral shear strain alone (I,K), fluid flow and surface shear strain (F,H), fluid flow and octahedral shear strain (J,L) for an imposed fluid flow of 100 µm/s and 10% compression**. Both trimetric and side views are shown.

**Figure 5:**
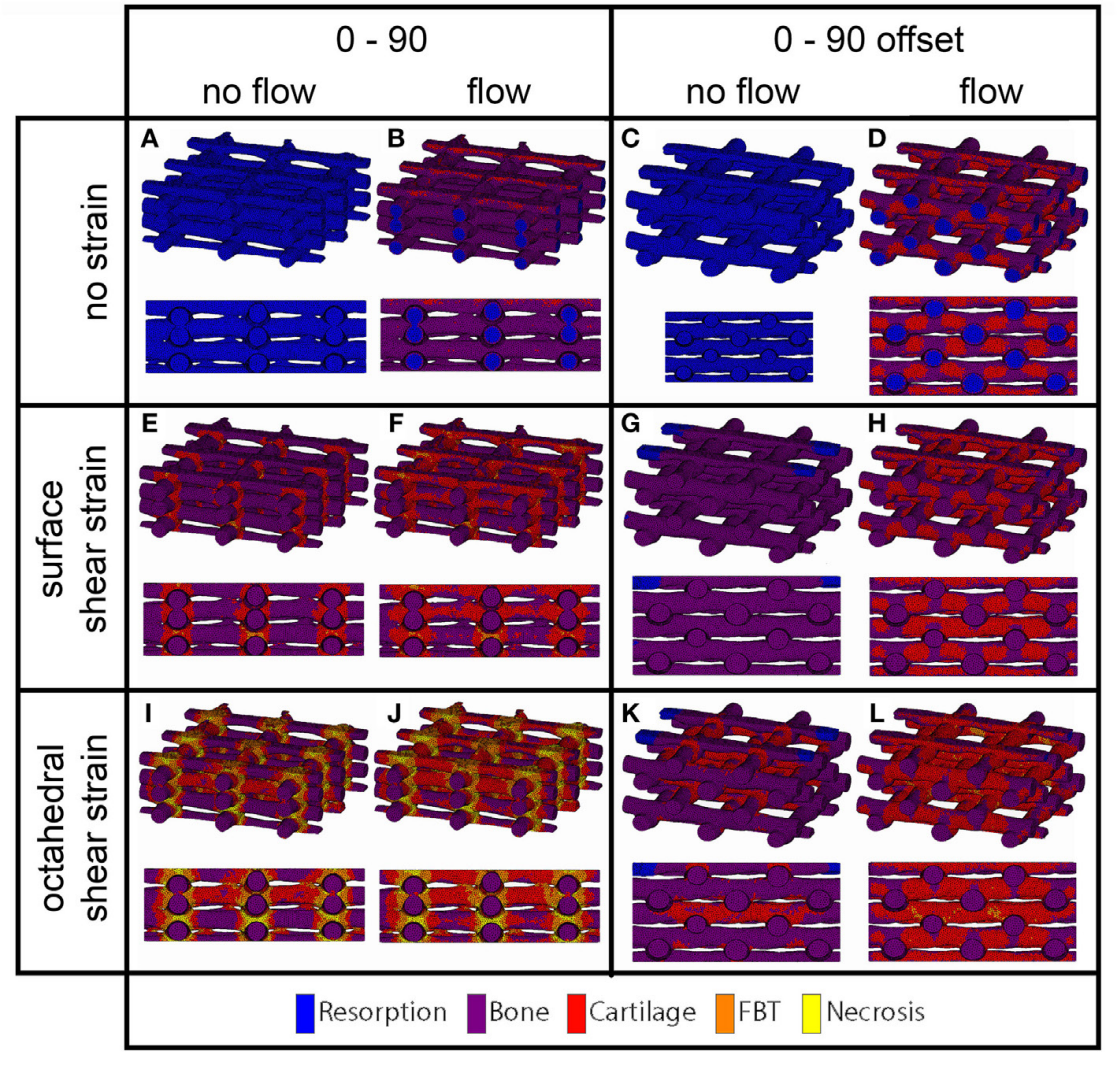
**Cell differentiation stimulus distribution for 0/90 (A,B,E,F,I,J) and 0/90 offset (C,D,G,H,K,L) architectures based on fluid flow alone (B,D), surface shear strain alone (E,G), octahedral shear strain alone (I,K), fluid flow and surface shear strain (F,H), fluid flow and octahedral shear strain (J,L) for an imposed fluid flow of 100 µm/s and 10% compression**. Both trimetric and side views are shown.

**Figure 6:**
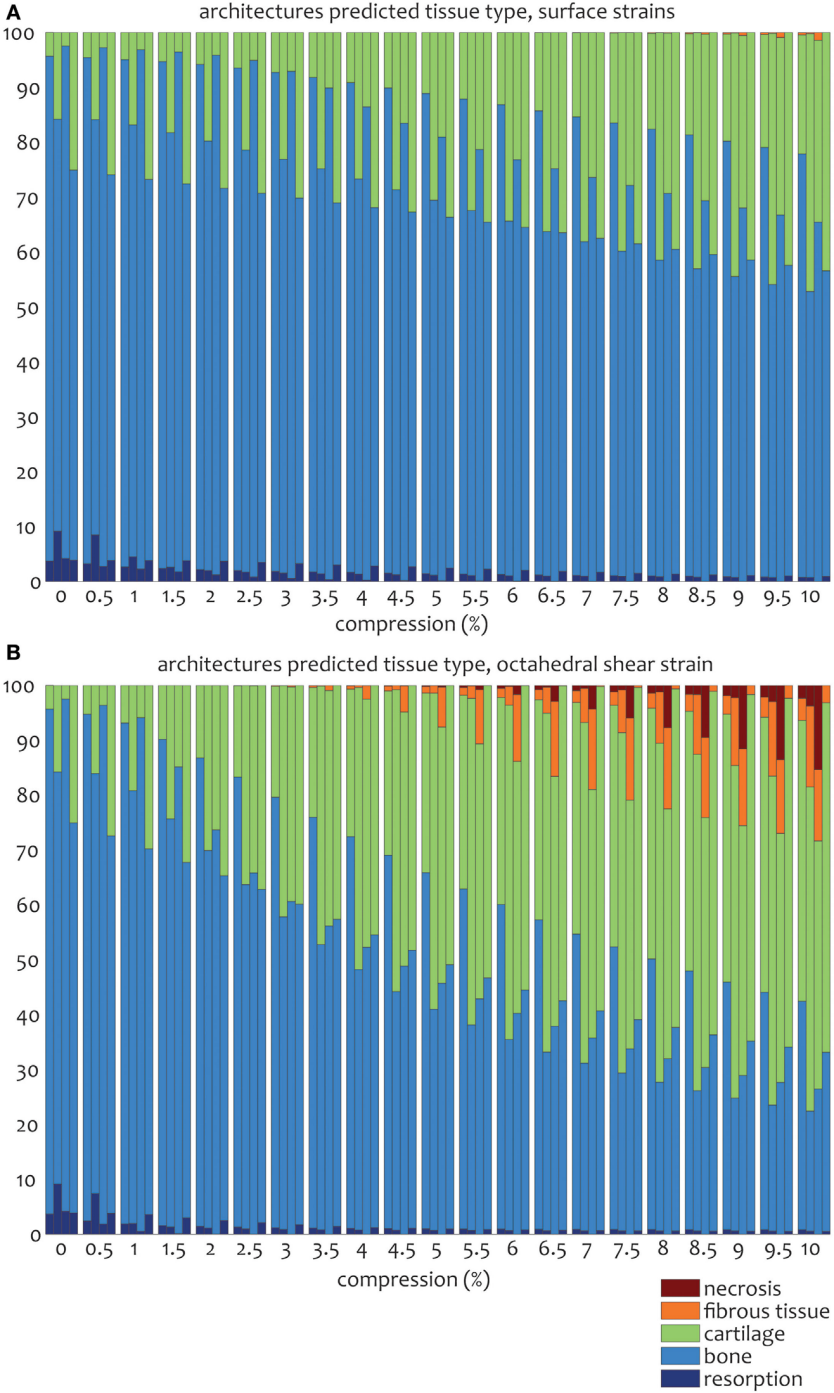
**Percentages of cell differentiation stimulus values on the surface of the scaffold for the 0/45 (left column), 0/45 offset (second to left column), 0/90 (third to left column), and 0/90 offset (fourth to left column) architectures based on the surface shear strain (A) and octahedral shear strain (B) for an imposed fluid flow of 100 µm/s and 10% compression**.

For the prediction of cell differentiation stimuli, a current mechano-regulation theory was applied relating the shear strain and fluid flow shear stress with cell differentiation. As expected given the methodology, prediction of the cell differentiation stimulus based on the surface shear strain with mechanical compression alone showed a profile similar to the strain distribution (Figures 2, 4 and 5). At the crossings of fibers, higher stimulus values were found resulting in cartilage for all but the 0/90 offset architecture, while in the 0/45 and 0/90 scaffolds patches of FBT were predicted (Figure 6). The 0/90 offset predicted bone formation and even some resorption for the 10% applied compression. However, the cell differentiation prediction based on the octahedral shear strain showed 63% cartilage in the 0/90 offset. For the 0/45 and 0/90 architectures, all stimulus values were increased and even showed considerable amounts of necrotic stimulus values.

The cell differentiation prediction stimulus based on fluid wall shear stress shows high similarities with the shear stress distribution (Figures 3–5). In the 0/45 and 0/90 scaffolds, bone and small amounts of cartilage for a fluid velocity up to 100 µm/s were predicted. However, for the 0/45 offset and especially for the 0/90 offset, cartilage was predicted already at 75 µm/s (data not shown) and showed a considerable amount of this tissue at 100 µm/s; 15 and 25%, respectively (Figure 6).

When the cell differentiation prediction was based on a combination of surface shear strain and fluid shear stress, the effects of 10% mechanical compression and 100 µm/s fluid velocity were additive. Within the 0/45 scaffold, the amount of cartilage increased from 4 to 21%, and small patches of FBT were predicted (4%). However, with the octahedral shear strain, besides more cartilage (51%), also necrotic tissue was predicted (2%) (Figure 6). The 0/45 offset showed a higher prediction for the amount of cartilage with the octahedral shear strain (46 vs 59%), while more fibrous (14%) and necrotic (3%) cell stimuli values were also predicted. For the 0/90 scaffold, more cartilage and FBT were predicted with the octahedral shear strain compared to the surface strain (33 and 1% vs 45 and 13%, respectively). With the octahedral shear strain, significant amounts of necrotic tissue (15%) were also predicted. In the 0/90 offset, the amount of cartilage predicted increased with both shear strains (from 25% without strain to 43% and 63% with surface strain or octahedral strain, respectively) while with octahedral shear strain, some FBT was also predicted (3%).

## Discussion

In tissue engineering, scaffolds designed for load-bearing applications should provide an environment that is capable of withstanding the forces that arise during the daily activities of a patient. In addition, the scaffold should provide differentiation stimuli to cells to ensure optimal tissue development. An important stimulus in this aspect is the dynamic mechanical environment that a scaffold can offer to the cells residing on the scaffold (Martin et al., 2004; Janssen et al., 2006; Koch et al., 2010; Sinha et al., 2015). By adapting the scaffold architecture, different stress and strain distributions can be achieved resulting in different cell differentiation stimuli for different architectures. In this paper, four scaffold designs were used, which only differed in the pore shape by using the same fiber diameter, spacing, porosity, and layer thickness. By changing the layer orientation, the effect of scaffold architecture on the stress and strain distribution, and subsequently the cell differentiation stimulus, was investigated. Measurements of fiber diameter, spacing, and layer thickness from the μCT scans showed similar values across all the architectures. As for the measured porosity, the values were slightly higher for the 0/45 architecture and slightly lower for the 0/45 offset architecture. Even though this will have an effect on the absolute strain values, strain distribution profiles are less likely to be directly affected by small differences in porosity values. The derived FEM from the respective scaffold showed the same porosities, indicating that a representative FEM was obtained for each architecture. Taken together, these results showed that the orientation angle between layers was the main variable across the four different scaffolds.

Using a device previously reported by Baas and Kuiper (2008), scaffolds were fixed at a compression of 10% and subsequently scanned. FEMs of the same complete scaffold were simulated to the same 10% compression. The simulated FEM was subsequently superimposed on the compressed scaffold scan in order to validate the simulation results (Figure 1). Small differences were visible mainly in the compression direction, which could be due to the scaffold not being positioned perfectly flat on the supporting surface during compression. Unfortunately, applying the same mesh mapping on both the compressed scaffold and FEM was not possible, and, therefore, no value for the correctness of the fit could be determined (Narra et al., 2015). However, comparison of the μCT scans with the FEA results showed a high degree of correlation, thus indicating that the results of the FEA are valid.

Upon mechanical compression of the scaffolds, strains mainly developed at the crossing of fibers. Both the magnitude of the strain and the surface area affected by the mechanical compression were influenced by the scaffold architecture (Figure 2). In the 0/90 and 0/45 scaffolds, the crossings of the fibers were located at the same position in each layer creating a vertical supporting column for the load. When looking at a 2 × 2 pore section of the scaffolds, the 0/90 architecture has nine of these vertical supporting columns, while the 0/45 architecture has six. Therefore, the 0/45 offers less support for the load which resulted in higher strain magnitudes in this architecture. Additionally, because of the columns, the surface area that was affected by the mechanical compression was confined to a small area around the crossing of the fibers. In the 0/45 offset and 0/90 offset architectures, vertical supporting columns were absent, resulting in a larger surface area affected by the mechanical compression. This was accompanied by strain magnitudes, which were lower in the case of the 0/90 offset architecture but higher in the case of the 0/45 offset architecture. Even though the higher values for the 0/45 offset architecture may be partially explained by the slightly lower porosity of this architecture in comparison with 0/45, this shows that the strain distribution is highly dependent on the scaffold architecture and that average strain values are inversely related to the fraction of the scaffold that actively carries the load. The dependence of strain profiles on the scaffold pore architecture is in line with previous literature (Sandino et al., 2008; Olivares et al., 2009; Milan et al., 2010). However, by investigating the strains that were developed on the scaffold surface, this study provides a better understanding of the exact mechanical signals that the cells attached to the scaffold surface experience.

In addition to strain values, the results of fluid flow analysis also depended on the pore shape (Figure 3). In all architectures, the fluid flow acted mainly at the sides of the fibers. Fibers located closest to the fluid inlet shielded the fibers in the lower layers from fluid flow and thus fluid shear stress. This was especially apparent in the 0/90 architecture. This means that the average shear stress values reported depend on the number of fiber layers included in the CFD model. For all architectures, the effective pore shape and size dictated the shear stress magnitude. The 0/90 architecture had effectively the largest pore size and, therefore, the lowest fluid shear stresses. On the other hand, the 0/90 offset had effectively the smallest pore size resulting in the highest shear stresses throughout the scaffold. For the 0/45 and 0/45 offset architectures, the offset of the fibers effectively reduced the pore size and shape, which increased the fluid shear stresses. Both scaffolds had shear stress magnitudes, which were in between the values for the 0/90 and 0/90 offset architectures. Comparing our results with literature, similar shear stress magnitudes with a 100 µm/s fluid velocity can be inferred (Milan et al., 2009). Also, Olivares et al. (2009) showed that the shear stress distribution and the magnitude of the stresses depended on the pore shape architecture, which corroborates our findings.

In order to determine the effect of different stress and strain distributions on the potential cell differentiation in the initial stage of cell attachment and differentiation on the surface of a tissue engineering scaffold, an adaptation of the mechano-regulation theory of Prendergast was used. The original theory combines octahedral shear strains with fluid flow shear stresses to predict cell differentiation (Lacroix et al., 2002). However, since the octahedral shear strain is a strain that acts in the volume of a material, the original model is not able to correctly predict the behavior of cells that are seeded on the surface of a material. This study shows that for all scaffold architectures, the octahedral shear strain is higher than the surface shear strain, which means that current models available in literature overestimate the mechanical signals that cells experience when seeded on a scaffold surface, resulting in an incorrect prediction of the cellular response.

The higher shear strain values seen from the mechanical compression at the crossing of the fibers resulted in the prediction of more cartilage formation and in some cases FBT at these locations. Large differences could be seen between the different scaffold architectures, depending on the strain magnitudes that were developed in the scaffolds. The influence of fluid flow on cell differentiation prediction correlated with fluid shear stress distributions. For the 0/90 offset architecture, the higher fluid shear stresses resulted in a larger amount of cartilage prediction, while predicted cartilage formation was lowest for the 0/90 architecture. The results from this study correlate well with a study by Olivares et al. (2009). At an inlet velocity of 100 µm/s, they reported predicted percentages for cartilage differentiation ranging from 18 to 45% depending on the scaffold architecture. The higher amount of cartilage predicted by Olivares compared to our study can be linked to the lower porosity of some of the architectures studied by Olivares, which ranged from 55 to 70% compared to the approximate 70% porosity for the architectures used in our study.

The combination of fluid flow and mechanical compression on cell differentiation stimulus prediction showed that the scaffold architecture had an effect on the relative contribution of both mechanical stimuli on cell differentiation stimulus values. Even though Figure 6 shows that stimulus values increase with an increasing applied compression for all geometries, this effect is relatively strong for the 0/90 geometry and relatively weak for the 0/90 offset geometry. This implies that in a geometry where compressive loads are distributed well over the scaffold volume, such as the 0/90 offset geometry, the relative effect of fluid perfusion on cell differentiation is increased. In a geometry where compressive loads are concentrated at specific locations due to supporting columns, high local stimulus values will arise as a result of compression, which means that the relative effect of compression on cell differentiation is high.

In this study, different software packages were used for the CFD and FEA simulations. Therefore, the effect of compression-induced fluid flow, as well as possible scaffold deformation due to fluid flow, cannot be taken into account. Although the fluid flow is low and can probably be neglected as a factor for scaffold deformation, the compression-induced fluid flow is likely to have a significant effect on cell differentiation predictions. When a total compression of 10% is applied, it is not unrealistic to assume that a significant fluid flow will occur due to this compression. Ideally, a coupled fluid-structure interaction model should be developed in order to address this aspect. However, these models are computationally very demanding, and it may be difficult to devise such a model for the complex geometries used in this study.

Finally, it should be noted that the scaffold architecture, and specifically the orientation of subsequent layers in scaffolds that are acquired using 3D fiber deposition, can have an effect on MSC cell differentiation even without external compression or fluid perfusion. A recent study by Di Luca et al. (2016) shows that in a gradient scaffold where the deposition pattern ranges from 0–90 to 0–15, squared pores support enhanced chondrogenic differentiation, whereas cells residing in the rhomboidal pores display enhanced osteogenic differentiation. This means that a model focusing solely on surface strains and fluid flow shear stresses is unlikely to provide a perfect prediction of cell differentiation and tissue development. Apart from that, the actual mechanical signals perceived by cells at the cellular level may be different from the values reported in this study. Zhao et al. (2015) used a multiscale model of an idealized CAD model and showed that the cellular placement on either the surface wall or bridging the scaffold pore has an effect on the signals experienced by the cell. Although the amount of cells placed were low and their morphology was not representative, the idea of using multiscale models to better estimate the local shear strain and stress perceived by cells is a valid one.

## Conclusion

The results of this study show that the scaffold architecture has a significant influence on stress and strain distributions. In particular, removing the possibility of load bearing with solid structures spanning from the top to the bottom of the scaffold, or even reducing the amount of these structures in a scaffold, has a great effect on the strain distribution. Additionally, the different architectures result in differences in the effective pore size and shape, which subsequently influence the fluid shear stress distribution. Therefore, by only changing the angle of orientation, varying stress and strain distributions can be exploited to design scaffolds for specific applications. Although in this study the material properties are based on the polymer PEOT/PBT, this methodology can be used for different biomaterials with different mechanical properties. The possibilities to design a scaffold with desired stress and strain distribution are therefore vast. Coupling these models with bioreactor studies to experimentally determine cellular differentiation within scaffolds subjected to specific fluid perfusion and compression regimes can provide valuable insights in the design of scaffolds and culture conditions for osteochondral, bone and cartilage replacements.

## Author Contributions

WH, YY, LM, and JR designed the experiments. WH and AD conducted the experiments. WH, LM, and JR analyzed data and wrote the manuscript. All the authors discussed the results and revised the manuscript.

## Conflict of Interest Statement

The authors declare that the research was conducted in the absence of any commercial or financial relationships that could be construed as a potential conflict of interest.

## Supplementary Material

Figure S1 **The four architectures of the scaffolds with 0/45 (A), 0/45 offset (B), 0/90 (C), and 0/90 offset (D) layers**.

Figure S2 **Circles of Mohr depicting the principal shear strain ϵ_1_, ϵ_2_ for the strains developed on the element face, which are used in Eq. 1 in this paper (A) and the principal strains ϵ_I_, ϵ_II_, and ϵ_III_ for the strains developed in the entire element which are used in Eq. 2 in this paper (B) lying on the surface of the model**.

Figure S3 **Shear strain distribution of the 0/90 offset architecture for surface shear strain (A) and octahedral shear strain (B)**. Both trimetric and side views are shown.
