# Supplementary figures and images for: Influence of Additive Manufactured Scaffold Architecture on the Distribution of Surface Strains and Fluid Flow Shear Stresses and Expected Osteochondral Cell Differentiation
Source: Front Bioeng Biotechnol. 2017 Feb 10;5:6. doi: 10.3389/fbioe.2017.00006 (PMC5300985; doi:10.3389/fbioe.2017.00006)

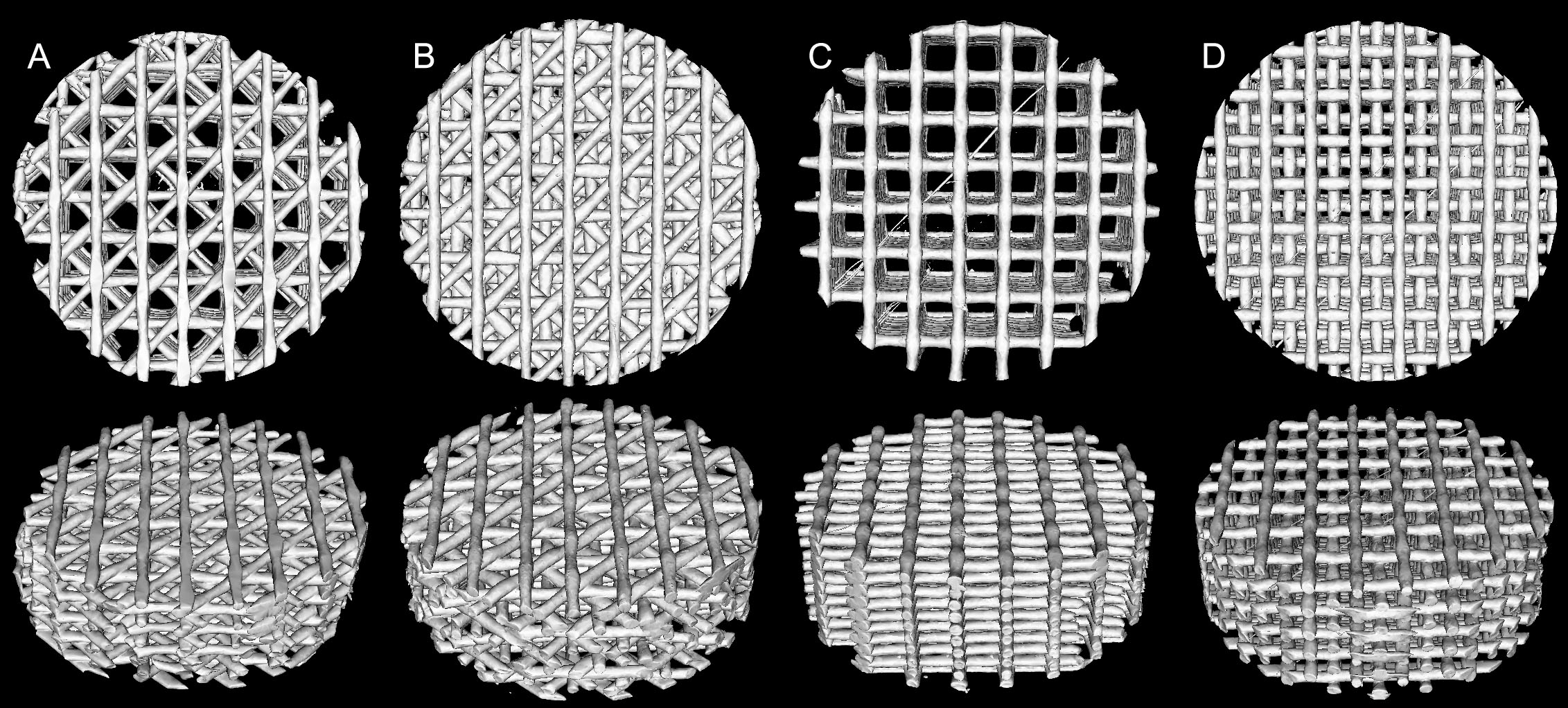

Supplement: Figure S1 — The four architectures of the scaffolds with 0/45 (A), 0/45 offset (B), 0/90 (C), and 0/90 offset (D) layers. [file image_1.jpeg]

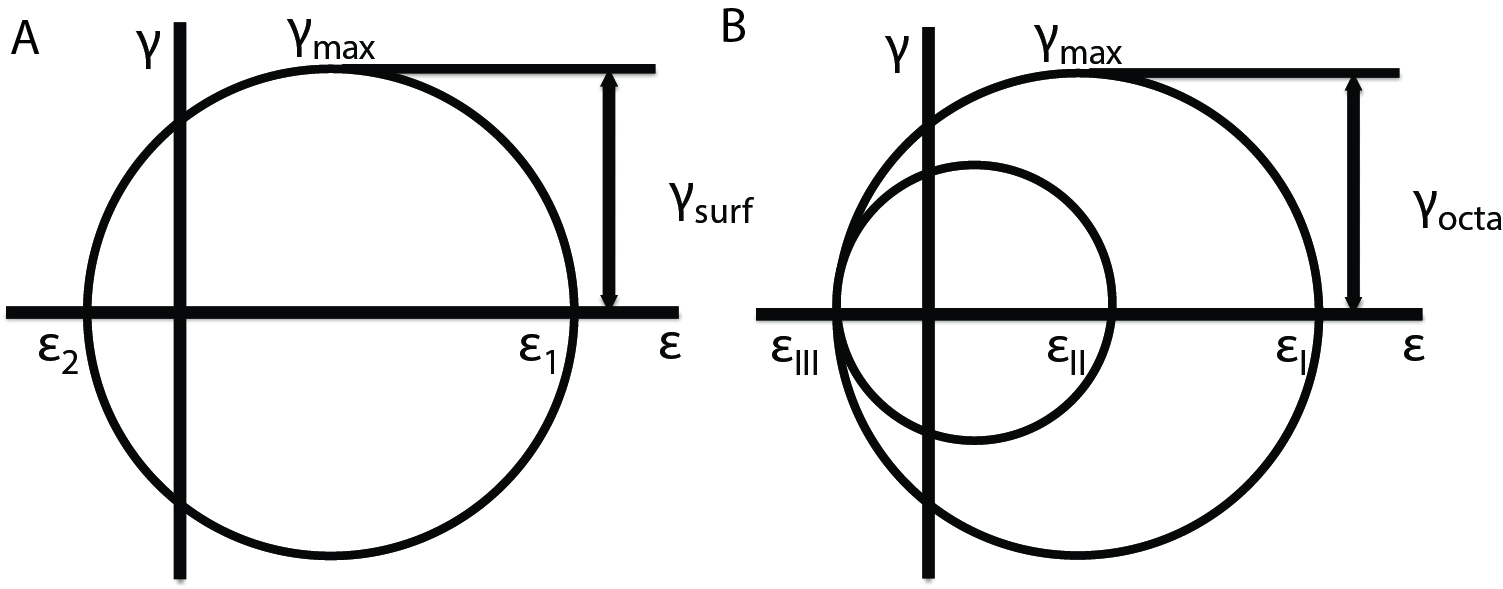

Supplement: Figure S2 — Circles of Mohr depicting the principal shear strain ϵ1, ϵ2 for the strains developed on the element face, which are used in Eq. 1 in this paper (A) and the principal strains ϵI, ϵII, and ϵIII for the strains developed in the entire element which are used in Eq. 2 in this paper (B) lying on the surface of the model. [file image_2.jpeg]

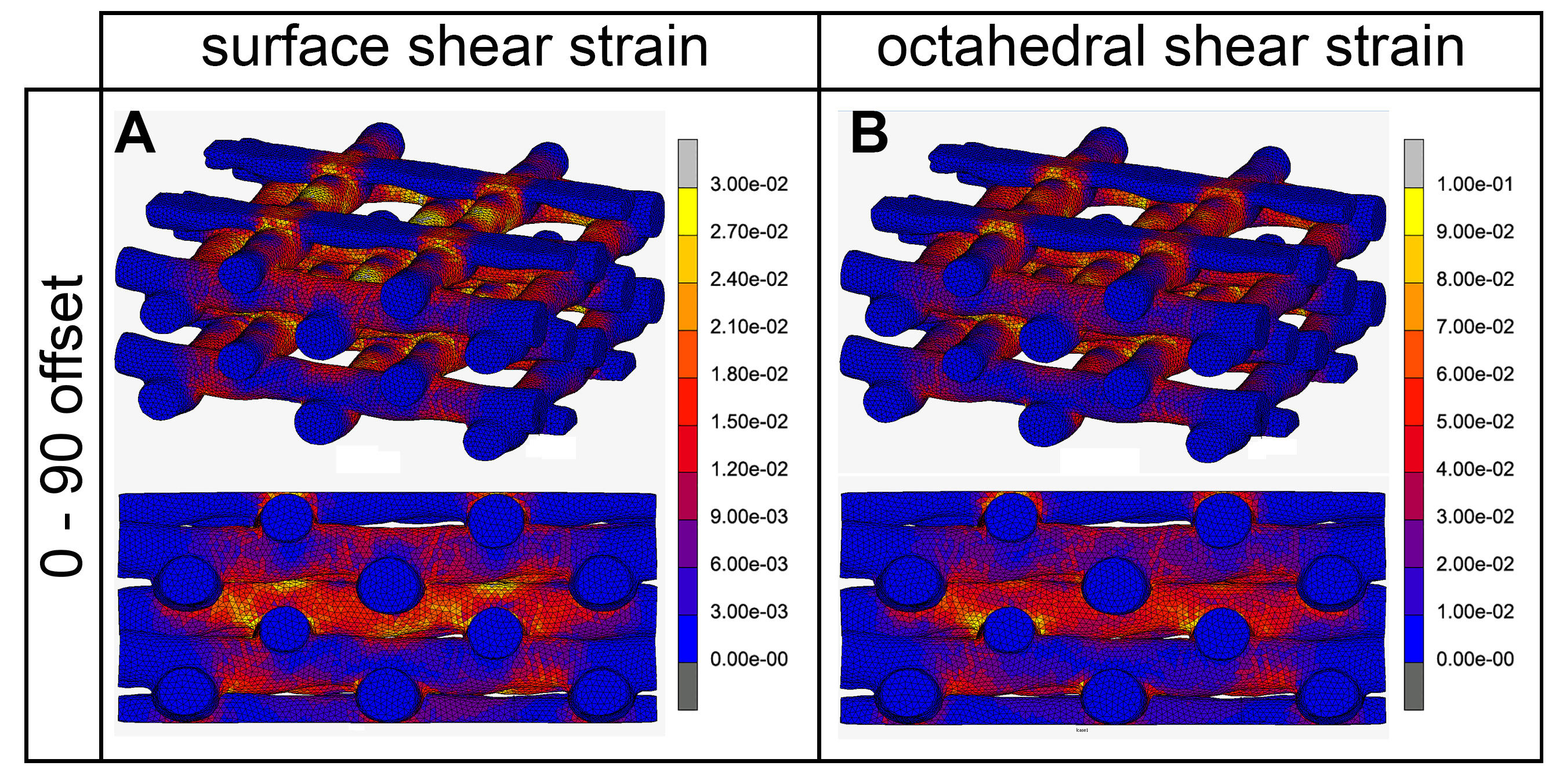

Supplement: Figure S3 — Shear strain distribution of the 0/90 offset architecture for surface shear strain (A) and octahedral shear strain (B). Both trimetric and side views are shown. [file image_3.jpeg]
